# Measurement of mismatch negativity in individuals: A study using single-trial analysis

**DOI:** 10.1111/j.1469-8986.2009.00970.x

**Published:** 2010-07

**Authors:** D V M Bishop, M J Hardiman

**Affiliations:** Department of Experimental Psychology, University of OxfordOxford, UK

**Keywords:** Mismatch negativity, Single-trial analysis, Reliability, Time-frequency analysis

## Abstract

Mismatch negativity (MMN) is measured by subtracting the averaged response to a set of standard stimuli from the averaged response to rarer deviant stimuli, and taking the amplitude of this difference wave in a given time window. This method is problematic when used to evaluate individuals, because there is no estimate of variance. We describe a new approach, in which independent components with high trial-by-trial variance are first removed. Next, each deviant response has the preceding standard response subtracted, giving a set of single trial difference waves. We illustrate this approach in analysis of MMN to brief tones in 17 adults. The best criterion for MMN combined *t*-test with an index of inter-trial coherence, giving significant MMN in 14 (82%) of individuals. Single-trial methods can indicate which people show MMN. However, in some clinically normal individuals there was no MMN, despite good behavioral discrimination of stimuli.

The auditory mismatch negativity (MMN) is an automatic neuronal response to change in events that occur close together in time. According to Näätänen (1992), a frequent sound in the environment (standard stimulus) is retained in auditory sensory memory, and other less frequent sounds (deviant stimuli) are compared with this memory such that any deviation from it will elicit a MMN response. Studies of the MMN have obtained robust results in group studies investigating questions such as the nature of auditory discrimination, the role of attention in auditory perception, and the impact of linguistic experience in determining responses to speech sounds (Näätänen, Paavilainen, Rinne, & Alho, 2007). Recent research has modified the traditional memory trace explanation of the MMN, recognizing the need to take into account the contribution of refractoriness in afferents responding to standards and deviants, and to account for the elicitation of MMN by deviations from predicted regularities when there is no repeating stimulus (Winkler, 2007).

Other studies have used the MMN as a window into auditory perceptual deficits in clinical groups, such as people with specific language impairment (SLI), or dyslexia (see Näätänen, 2003, for review). Quite simply, if the brain does not detect the difference between standard and deviant sounds, then no MMN should be observed. This makes it a useful index for testing theories that postulate auditory perceptual deficits as the basis for developmental disorders. Such theories propose that, even if peripheral hearing is normal, the ability to distinguish certain sound features, such as frequency or temporal characteristics, may be impaired. The rationale that is typically given is that MMN gives a more direct indication of the brain's ability to discriminate stimuli than behavioral measures, which may be influenced by attentional or motivational factors. In this clinical area, however, findings have been mixed. Bishop (2007) reviewed studies of children with SLI or dyslexia, and found many failures to replicate from one study to another. Although differences in study samples, stimulus materials, and analytic methods could account for some variation, a more general issue is the finding that MMN and behavioral testing often fail to correspond. If a participant shows an MMN but fails a behavioral discrimination test, this confirms that the earliest stages of sound processing in the brain are intact. However, the converse pattern is more problematic to interpret, i.e., individuals who apparently lack an MMN but who can discriminate the stimuli behaviorally. Several studies have described such cases in normal samples (Dalebout & Fox, 2000, 2001; Kurtzberg, Vaughan, Kreutzer, & Fliegler, 1995; Uwer & von Sucholdoletz, 2000). This raises doubt about the utility of the MMN as an indicator of auditory discrimination by the brain in clinical contexts. Because MMN is typically estimated from a single measure of mean or peak amplitude over a given time window, it is hard to know whether such cases are ‘true negatives’ or reflect error of measurement in an imperfectly reliable measure. The test-retest reliability of the MMN has ranged from .3 to .7 in studies where individuals are tested on more than one occasion (Escera & Grau, 1996; Escera, Yago, Polo, & Grau, 2000; Kurtzberg et al., 1995; Lew, Gray, & Poole, 2007; Pekkonen, Rinne, & Näätänen, 1995; Tervaniemi et al, 1999; Uwer & von Suchodoletz, 2000;). Reliability is similar for the magnetic counterpart, MMNm (Tervaniemi et al, 2005).

A study by McGee, Kraus, and Nicol (1997) is salutary when considering assessment of the MMN in individuals: they compared various methods for assessing the MMN, and found that expert raters were prone to label difference waveforms as showing MMN even when the standard and deviant were identical. A range of methods has been used to try to optimize measurement of the MMN, with some preferring use of mean amplitude in a given time window, and others using peak latency or mean amplitude over a window adjusted for the individual's peak latency (Sinkkonen & Tervaniemi, 2000). However, none of these approaches addresses the issue of within-subject variability in the waveform. Ponton, Don, Eggermont, and Kwong (1997) argued for use of the integrated MMN, in which the amplitude of each time point is added to the preceding time-points. The averaged integrated MMN can then be compared to integrated MMNs from a set of individual standard trials, selected at random, giving a method that does take into account trial-by-trial variation. This method has not, however, been widely adopted by other researchers, perhaps because it is computationally intensive. Furthermore, it requires the experimenter to specify a time point at which the averaged deviant integrated amplitude is compared with the subset of standard amplitudes, and thus does not overcome difficulties arising when comparing individuals or groups who may have different latencies of mismatch response. Ponton et al. (1997) presented sample data from one deaf and one hearing participant, but did not provide information about the proportion of normally hearing individuals who obtained a significant MMN using this method. It therefore remains unclear how useful it would be in clinical applications, where lack of mismatch response is to be regarded as an index of abnormality. Another method that considers variability as well as mean amplitude of responses was considered by McGee et al. (1997), who divided a dataset into sub-blocks, each containing 25 deviant stimuli. For each sub-block, an MMN was computed, giving a set of 8–10 estimates of MMN for each participant. Point-to-point *t*-tests were then conducted to identify intervals that were significantly below zero. However, in their study of responses to speech stimuli by children, the method was not very effective in discriminating true mismatch sessions from those where the same stimulus was used for standard and deviant.

Here we present a single-trial analysis of data from a sample of 17 adults, and demonstrate that this approach allows us to determine when an individual participant has a reliable MMN. Conventional analysis of a subset of the data reported here was presented by McArthur, Bishop and Proudfoot (2003). We used the EEGLAB v6.03b software package (Delorme & Makeig, 2004) to introduce several novel steps in the re-analysis of these data, as illustrated in Figure 1, and described more fully below.

**Figure 1:**
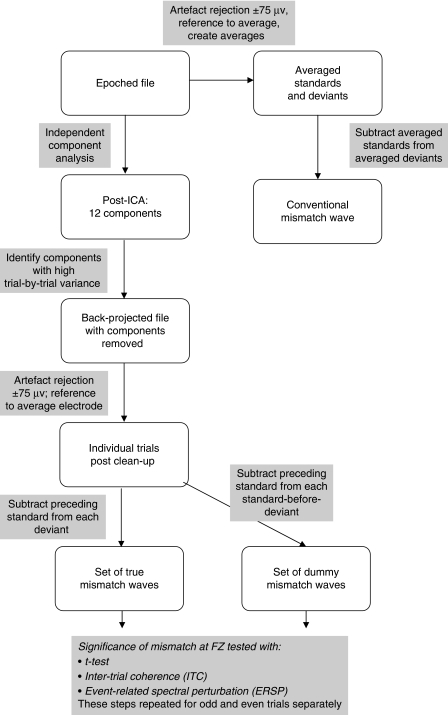
Flowchart showing stages of processing.

## Methods

### Behavioral Assessment of Frequency Discrimination Threshold

Auditory frequency discrimination was assessed using a three-interval, two-alternative forced choice method of threshold estimation. The task was to indicate which of two tones (A or B) had a higher frequency than a standard 600-Hz 25-ms pure tone (X). The frequency of the different tone was varied adaptively to converge on a 79% correct threshold. The three stimuli in a trial were separated by 500-ms silent intervals and presented in the order AXB, with the different tone being in position A or B at random. For further details, see McArthur and Bishop (2005).

### Electrophysiological Methods

**Figure 2:**
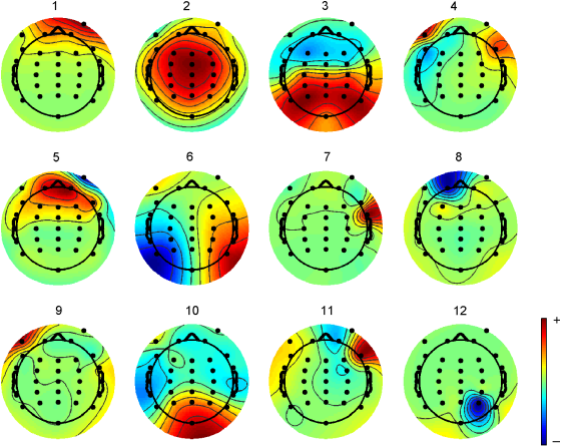
Illustrative ICA decomposition for subject #1. Components 1, 4, 5, 7, 9, and 12 were rejected on the basis of high standard deviation of mean absolute amplitude across trials.

Experimental event-related potential (ERP) methods are described in detail by McArthur et al. (2003) and are summarized in Table 1. Offline analysis was conducted using EEGLAB software (Delorme & Makeig, 2004). Artefact rejection proceeded in two phases. First, the data were subjected to independent components analysis using the EEGLAB function “runica.m” to extract 12 components, with the PCA option first applied to reduce the rank of the data and speed up the analysis. Twelve components were specified on the basis of exploratory principal components analysis indicating that higher rank components did not account for more than 1% of variance. An additional routine was written to identify components with high levels of trial-by-trial variance: For each trial, mean absolute amplitude was computed across all time points, and the standard deviation (SD) of these mean values was computed across trials. A cutoff was determined by trial and error. For 16 of 17 participants, rejection of components with SD greater than 0.8 μV gave good agreement with subjectively judged component rejection, in that rejected components did not include those with fronto-central distribution, but were components with high loadings from eye channels, or which showed highly focal activity suggestive of artefact, as illustrated in Figure 2. For the remaining participant (#15), all components were rejected by this criterion, and so the cut-off SD was incremented in steps of .1 until some components were retained, giving a cut-off SD of 1.2 μv. After component removal, data were re-referenced to the average of all electrodes, to allow for visualization of the mastoid, and trials with activity greater than ±75 μV were excluded. Re-referencing and the ±75 μV rejection criterion were also applied to the original waveforms, so that the impact of component removal by ICA could be assessed.

**Table 1:** Details of Experimental Paradigm

Participants*	17 normal hearing adults aged 19 to 50 years
Stimuli	Condition 1: 600 Hz pure tone standard; 700 Hz deviant
	Condition 2: 700 Hz pure tone standard: 600 Hz deviant
Stimulus duration	25 ms
Stimulus intensity	80 dB SPL
Deviant frequency	15%
SOA	Randomly jittered between 870 and 970 ms
Total trials	1200 trials divided into 10 blocks of 200 stimuli selected randomly from condition 1 or condition 2
Recording system	Synamps
Electrode montage	10–20 System: FP1, FP2, F7, F3, FZ, F4, F8, FT7, FC3, FCZ, FC4, FT8, T7, C3, CZ, C4, T8, FP7, CP3, CPZ, CP4, TP8, P7, P3, PZ, P4, P8, OZ, left and right mastoids, and VEOG and HEOG channels
Reference	Online linked mastoids; offline average reference
Ground	Intermediate between FPZ and FZ
Amplification	20,000
Sampling rate	250 Hz
Online filter	0.01–70 Hz (SynAmps), plus 50 Hz notch filter
Epoch length*	−200 to 500 ms
Artefact rejection*	By ICA (see text) followed by rejection of trials with activity exceeding ± 75 μv

* *Note*: In original analysis by McArthur et al. (2003), baseline was 50 ms duration, and an ocular artefact rejection algorithm was used plus rejection of trials with activity exceeding ± 150 μV. Two participants from McArthur et al. were found to have had timing errors in stimulus presentation and are excluded here.

**Figure 3:**
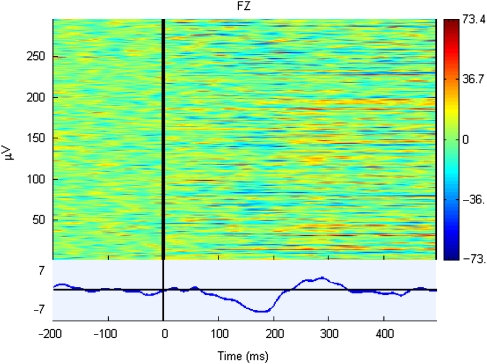
Sample ERP image for a set of difference waves (subject #1), formed by subtracting the preceding standard from each deviant. Each difference wave corresponds to a horizontal line, with color indicating amplitude (red positive and blue negative) over the time range indicated on the horizontal axis. The mean amplitude of the difference wave is shown in the lower panel.

For single-trial analysis of difference waves, we focused on electrode FZ, where the difference response was maximal. The preceding standard trial was subtracted from each deviant trial, to give a set of difference waves as shown in the illustrative ERP image in Figure 3. For each participant, a *t*-test was conducted at each time point to compare the distribution of amplitudes with zero. This procedure is comparable to the procedure adopted by Guthrie and Buchwald (1991) for analyzing sets of averaged difference waves from a group of participants, but in this case the set of difference waves correspond to single trials from one participant. Guthrie and Buchwald noted that, because the data at different time points are not independent, conventional significance levels of *t*-values are misleading, but they recommended that, where consecutive points in a series of *t*-values exceeds a significance level of .05, then the difference is likely to be reliable. The length of the required series will depend on the autocorrelation between consecutive data points, whether a directional prediction is made (enabling use of one-tailed tests) and the number of points in the range of interest. To minimize the chance of generating spurious significance, we used a predefined period from 100 ms to 232 ms (33 points) post-onset as the time window for evaluating significance of consecutive *t*-values, with one-tailed tests.

The independent component analysis (ICA)-rejection method led to the retention of many more trials than would be achieved by the more conventional analysis of rejection of large artefact by employing a voltage window (see below). Thus, it was possible to consider the reliability of the *t*-test procedure by dividing the set of difference waves into odd and even trials and applying the *t*-test procedure to the two data sets.

### Time-Frequency Analysis

**Figure 4:**
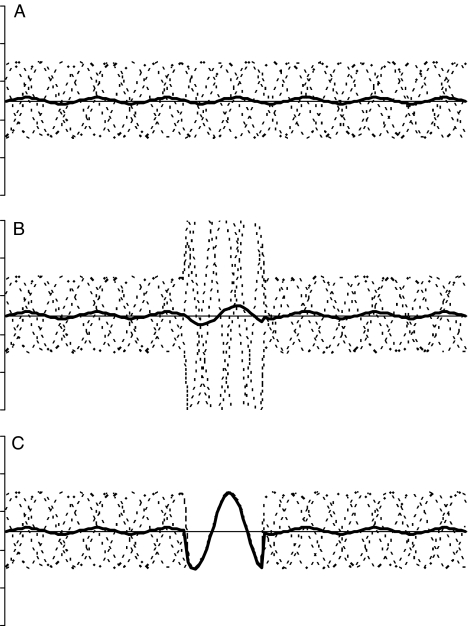
Illustration of logic behind time-frequency analysis. Panel A depicts five sine waves (dotted lines), with time on x-axis and amplitude on y-axis, (arbitrary units) at constant amplitude in random phase, with the bold line depicting the averaged waveform. Because the component waves are out of phase, they cancel out, so the averaged waveform is nearly flat. Panel B depicts the same data but with a time period during which the amplitude of the individual sine waves is multiplied by 3. The averaged waveform shows a corresponding small increase in amplitude. Despite a large increase in power, the averaged waveform would remain flat if the phases of the component waves completely cancelled out. The index of ERSP would, however, detect the increased amplitude. Panel C depicts the situation where there is a time period during which the component waveforms are reset to be in phase, without any change in amplitude. The average waveform now shows a clear peak. The measure of inter-trial coherence is sensitive to this synchronization of phase, even if amplitude does not change.

Makeig, Debener, Onton, and Delorme (2004) noted that a peak from an averaged ERP could arise because of an increase in power, and/or because of event-related phase-locking between trials (see Figure 4). They describe two methods of time-frequency analysis which are intended to help distinguish these underlying factors (see also Roach & Mathalon, 2008). The first, the event-related spectral perturbation (ERSP), is computed by first calculating the amplitude spectrum during the baseline period prior to stimulus presentation. The epoch is then divided into brief, overlapping data windows, and a moving average of the amplitude spectra of these is derived. The spectral transforms are normalized by dividing them by their respective mean baseline spectra, to give a measure in decibels (dB) of event-related changes in power (Makeig, 1993). It is logically possible to have significant ERSP even if the ERP does not reveal a peak, if amplitude enhancements at a given frequency are out of phase and so cancel each other out, as discussed in relation to panel B of Figure 4. Thus, it is of particular interest to consider ERSP for individuals who do not appear to show an MMN. For the current dataset, ERSP in dB was computed by the ‘timef’ function from EEGLAB using standard wavelets with one cycle per analysis wavelet. Only the lowest frequency band, corresponding to the theta range, with mid-frequency at 7 Hz, is considered here, since preliminary analyses indicated that the difference waves contained little power in higher frequency ranges. Bootstrap statistics were used to determine intervals where ERSP was greater than in the baseline interval, using a .05 level of significance.

The second index, inter-trial coherence (ITC), with range 0 to 1, provides a measure of the degree of phase-locking between single trials in specific frequency bands, with range 0 to 1. In Figure 4 panel C, ITC is high during the intermediate portion where a clear signal emerges in the grand average, even though there is no increase in ERSP. ITC was computed using the same ‘timef’ function as for ERSP, again with a focus on the theta frequency range, and adopting a .05 level to identify regions where coherence was significantly greater than in the baseline interval.

## Results

### Artefact Removal Using ICA

**Figure 5:**
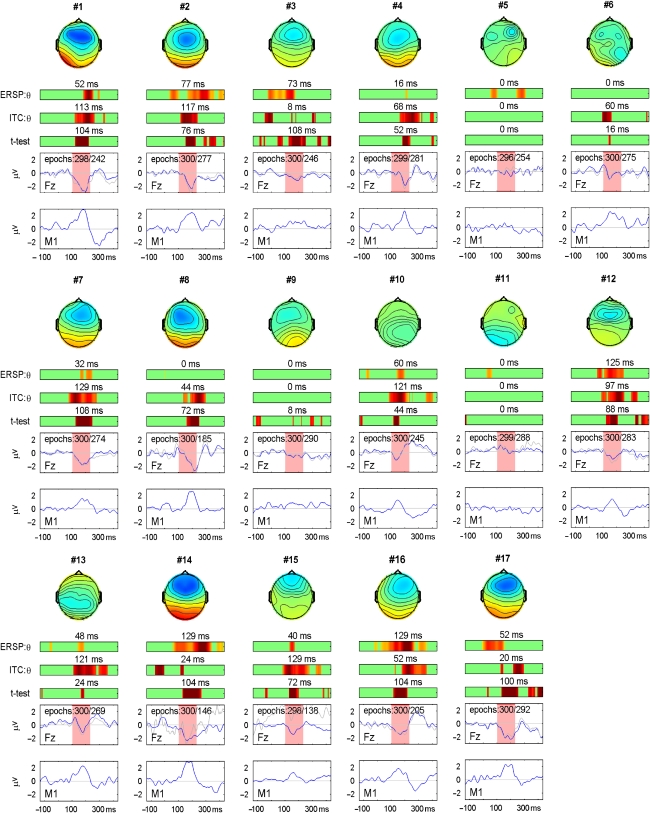
Results for individual participants (numbered) with 2D head plots indicating mean amplitudes during the interval 100–230 ms, color-range from −3 μV (blue) to +3 μV (red). Lowest panels show difference waveforms for each participant showing mean amplitude in μV on y-axes and time in ms on x-axes. Blue line for FZ panel shows data after removal of components with high variance, gray line shows original data after removal of trials with activity outside the range ±75 μV. Number of epochs is shown for blue line, then gray line. Color bars show regions of significance at .05 level for (a) *t*-test comparing mean amplitude with zero; (b) ITC relative to baseline, and (c) ERSP increase relative to baseline. The longest consecutive period of significance in the interval 100–232 ms post-onset is shown above each color bar.

Total number of difference-wave trials was 300. After rejection of components with high variance and recomputation of the back-projected ERP from remaining components, no participant had more than five trials removed by applying a criterion of ±75 μV. In contrast, when ICA component rejection was not used, the average number of trials rejected by this criterion was 53 (range 8–262; see Figure 5).

### Single-Trial Analysis of Difference Waves

The two lower panels for each participant in Figure 5 show the averaged waveforms for an individual participant at FZ and the averaged mastoid for both the original dataset, after applying ±75 μV artefact rejection (gray line), and the dataset after artefact rejection by ICA (blue line). It is noteworthy that, in the majority of cases, both artefact rejection methods lead to a similarly shaped waveform; however, the larger number of contributing trials with the ICA rejection method gives a more reliable estimate of amplitude. Exceptions are participants #14 and #15, where the waveforms using traditional amplitude-based artefact rejection are much noisier, because the ±75 μV criterion led to rejection of more than 50% of trials.

Figure 5 also shows, for each participant, regions where one-tailed *t*-test of the MMN (significantly below zero) is significant at .05 level, with the duration of the interval of significance in the range 100–232 ms given above the color bar. With *t* set at 1.65 (.05 one-tailed), if we estimate autocorrelation between successive data-points in the waveform as .9, then the simulations of Guthrie and Buchwald (1991) indicate a minimum sequence length of 32 ms (8 consecutive data points at 250 Hz sampling rate), is significantly different from chance expectation for an interval spanning 130 ms. Twelve of seventeen participants (70%) had a significant MMN on this criterion: #1, #2, #3, #4, #7, #8, #10, #12, #14, #15, #16, and #17. In all cases, those meeting criterion on *t*-test also had a significant interval of ITC and/or event-related spectral perturbation (ERSP). Furthermore, the mastoid traces for these participants show reversal of the waveform in the MMN interval, and inspection of the 2D head plots indicates a fronto-central focus of negativity in all participants with MMN, except #10 and #13, both of whom show more posterior activity. Of the five participants who do not meet *t*-test criteria for MMN, two (#6 and #13) show mastoid reversal and would meet criteria if ITC rather than *t*-test were used. Participant #9 shows a hint of an MMN-like response, but it is small in magnitude and does not achieve significance. A further two participants (#5 and #11) show little evidence of an MMN, either on the numerical criteria from *t*-test, ITC, or ERSP, or in terms of topography of response. The five participants who did not show significant MMN on *t*-test did not differ from the other participants on a measure of signal-to-noise ratio of responses to standards. This was assessed by dividing the standard deviation of amplitudes in mean response over interval 100–300 ms by the standard deviation of amplitudes in mean responses in the baseline (mean for those with MMN was 4.00, *SD*=2.52, and for those without MMN mean 4.91, *SD*=2.26).

The question arises as to whether somewhat laxer criteria could be used for MMN detection, or whether this would simply generate false positive results. To consider this question, the dummy files, formed by subtracting the preceding standard from each standard-before-a-deviant, were subjected to the same analysis. The plots are shown as Supplementary material in the online version of this article. Two (12%) of the 17 participants (#5 and #10) had significant *t*-values in the 100–232 ms interval lasting more than 32 ms, and both showed a topography resembling that of MMN, with frontocentral negativity and mastoid reversal. Since these waveforms were made by subtracting responses to identical stimuli, these must be false positive MMNs. The main feature distinguishing them from true MMNs was the lack of a sharp peak to the response at either FZ or the mastoid. It may also be noted that, whereas intervals of significant ITC were rare in the dummy plots, there were six instances of significant ERSP of duration of 32 ms or more, suggesting that ERSP is not a reliable indicator of a true MMN (at least when measured using the parameters specified above).

The analysis of deviant-minus-standard mismatch responses was repeated just for odd and even trials for each participant to establish reliability of findings (see Figures S3 and S4 in Supplementary Material). On the basis of findings with all trials, the criterion for MMN was changed to require an interval of 32 ms significance in the interval from 100–232 ms for *either t*-test *or* ITC. Ten participants (#1, #2, #7, #8, #10, #12 #14, #15, #16, and #17) met the criterion for significance in both halves, and three (#3, #4, #13) met the criterion in one half, with a shorter interval of significance in the other half. The four remaining participants (#5, #6, #9, and #11) were all cases who had given ambiguous or lack of evidence of MMN when all 300 trials were considered.

This analysis gives confidence that, for the majority of individuals, the MMN can be reliably detected using this method, provided 300 deviant trials are available, but the situation is less satisfactory when the number of trials is halved.

### Reconsideration of Participants Who Did Not Show Significant MMN on t-Test

Where a participant did not show significant MMN, even when all 300 trials were considered, the question arises as to whether a true MMN was inadvertently removed from the data by selection of specific components for analysis, or whether there is a genuine absence of response. For the five participants (#5, #6, #9, #11, and #13) who did not show a significant *t*-test for a consecutive 32 ms interval, the original 12 component ICA solution was inspected, with components selected subjectively to focus on those with a fronto-central distribution. In no case did this provide evidence of a significant MMN.

### Relationship of MMN to Behavioral Frequency Discrimination Thresholds

The mean frequency discrimination difference limen was 5.8 Hz (*SD*=3.42 Hz), with all participants obtaining thresholds well below the 100 Hz difference between standard and deviant tones. Thus, participants who did not show MMN had excellent ability to discriminate the tones used as standard and deviant. Pearson correlations were computed between measures of size of MMN (mean and peak amplitude over the interval from 100–232 ms, peak latency, and duration of significant *t*-values) and frequency discrimination thresholds. These were all non-significant (*r*=.38, .24, .06, −.19, respectively), although it should be noted that this study is underpowered for detecting weak correlations.

## Discussion

We report a novel analysis of MMN data, which makes it possible to identify reliable MMN responses in individuals. A key feature of the analysis was use of independent component analysis (ICA) to reduce artefact. By removing components with high trial-by-trial variance, it was possible to retain virtually all trials in the analysis. Note that this ICA was done on the full ERP dataset, using an automated procedure, with all standards and deviants combined, prior to computation of difference waves. It is more common to use subjective judgment to identify components for removal, but our concern was that this could introduce unwitting bias. A second feature was the use of single-trial analysis with difference waves formed by subtracting the preceding standard from each deviant waveform. This analysis used the back-projected data obtained after removing rejected components. This meant that for each participant there was a set of around 300 difference waves, which could then be tested using conventional *t*-test methodology to identify intervals that differed significantly from zero. A third feature was the use of time-frequency analysis in combination with more conventional amplitude criteria for identifying periods of significant mismatch. The ITC proved useful as a confirmatory indicator of significant mismatch in cases where the duration of the *t*-defined mismatch interval fell short of significance. Combining information in this way runs the risk of achieving spurious ‘significance,’ but confidence in this approach was provided by comparing the frequency of significant findings from genuine mismatch difference waves to that found with dummy difference waves formed by subtracting one standard from another. This gave reassurance that the method rarely over-identifies random fluctuations as MMN.

Although the goal of this study was practical and methodological, it is worth commenting on our finding that ITC is a more reliable signature of MMN than event-related spectral perturbation (ERSP). Although the distinction between ITC and ERSP has been argued to be key in distinguishing between phase resetting and standard accounts of ERP generation, this has been challenged by simulations by Yeung, Bogacz, Holroyd, and Cohen (2004), and Yeung, Bogacz, Holroyd, Nieuwenhuis, and Cohen (2007). They found that features thought to be indicative of phase resetting could be observed in simulated phasic peaks embedded in noise, because of ringing artefacts, and did not differ appreciably from those seen in data generated by synchronizing oscillatory activity. High ITC cannot, therefore, be regarded as a signature of a phase-resetting mechanism as the basis of MMN, although it is compatible with such an account. Time-frequency analysis of the MMN was undertaken by Fuentemilla, Marco-Pallarés, Münte, and Grau (2008), but these authors adopted a different analytic approach; in an initial analysis, instead of focusing on individual differences between participants they pooled responses from 16 adults. Rather than analyzing ITC and ERSP of the difference waves, as was done here, they compared the difference in ITC and ERSP of the standard and deviant stimuli. Subsequently they examined ITC and ERSP at different electrodes in individuals, and found that frontal components of the MMN were formed by an increase in both ITC and ERSP, whereas temporal components of the MMN were formed by phase alignment alone. Our analysis, however, suggests that the MMN is described best by changes in ITC, since changes in ERSP were inconsistent across subjects and seen even when there was no mismatch. It should be noted, however, that the typical baseline length used in MMN studies is relatively short for ERSP analysis.

If the MMN is to fulfil its promise as a clinical indicator of abnormal auditory processing (Csépe & Molnár, 1997), then we need paradigms that reliably give MMN in individual participants in non-clinical samples. That was not the case for the paradigm used here: in a sample of typical adults, we found that 14 of 17 (82%) showed significant durations of mismatch on a *t*-test and/or ITC when a full dataset based on up to 300 deviant responses was available, but this dropped to 11–13 cases (64–76%) when the number of trials was halved. Although this means that the majority of individuals gave convincing evidence of MMN with the larger dataset, around 18% of these normal adults did not. Furthermore, with dummy datasets created by subtracting a standard from the prior standard, our criterion for MMN identified 2/17 (12%) false positives. This does not rule out the use of MMN as a clinical tool: where a significant MMN is found, this is strong evidence that auditory processing or language comprehension is not due to a failure in early neural detection of differences between the sounds used as standard and deviant, particularly when the difference wave shows a clear peak that reverses at mastoids. However, failure to find significant MMN cannot be taken as an indication of abnormal functioning of auditory systems.

It is possible that a higher proportion of individuals would show significant mismatch in a different paradigm. Although the standard and deviant tones used in this study were readily discriminable (600 versus 700 Hz), they were only 25 ms in duration, briefer than is usually used for MMN studies. Nevertheless, it is noteworthy that among those without a significant MMN were some individuals who showed not even a trend for a difference in the predicted direction over the critical interval, despite showing clear ERPs to standards and deviants, and good ability to discriminate the stimuli on a behavioral test. It may be that the response is simply not evident in some individuals, perhaps because of atypical underlying gyral configurations, which would mean that generators of auditory responses might be oriented so as to preclude measurement at the scalp surface. It is also possible that clearer evidence for a mismatch response might be found if time-frequency analysis were extended beyond the theta range. Our data suggest that this method of analysis has promise as a clinical tool, because it can identify MMN at the individual level, but further studies are needed to discover whether it is possible to develop a paradigm that will show significant mismatch in all normal individuals for auditory dimensions of interest.

A final point to note is that there is no reason to restrict application of the methods described here to the MMN. Other components of clinical interest, such as P300, N400, ERN, could be analyzed in comparable fashion.

## Supporting Information

**Figure S1.** Mismatch plots: deviant minus preceding standard.

**Figure S2.** Dummy plots: standard minus preceding standard.

**Figure S3.** Mismatch response, Even trials.

**Figure S4.** Mismatch response, Odd trials.
