# Supplementary material for: Measurement of mismatch negativity in individuals: A study using single-trial analysis
Source: Psychophysiology. 2010 Jul;47(4):697–705. doi: 10.1111/j.1469-8986.2009.00970.x (PMC2904495; doi:10.1111/j.1469-8986.2009.00970.x)
Supplement: Supplementary file 1 [file psyp0047-0697-SD1.ppt]

## Slide 1
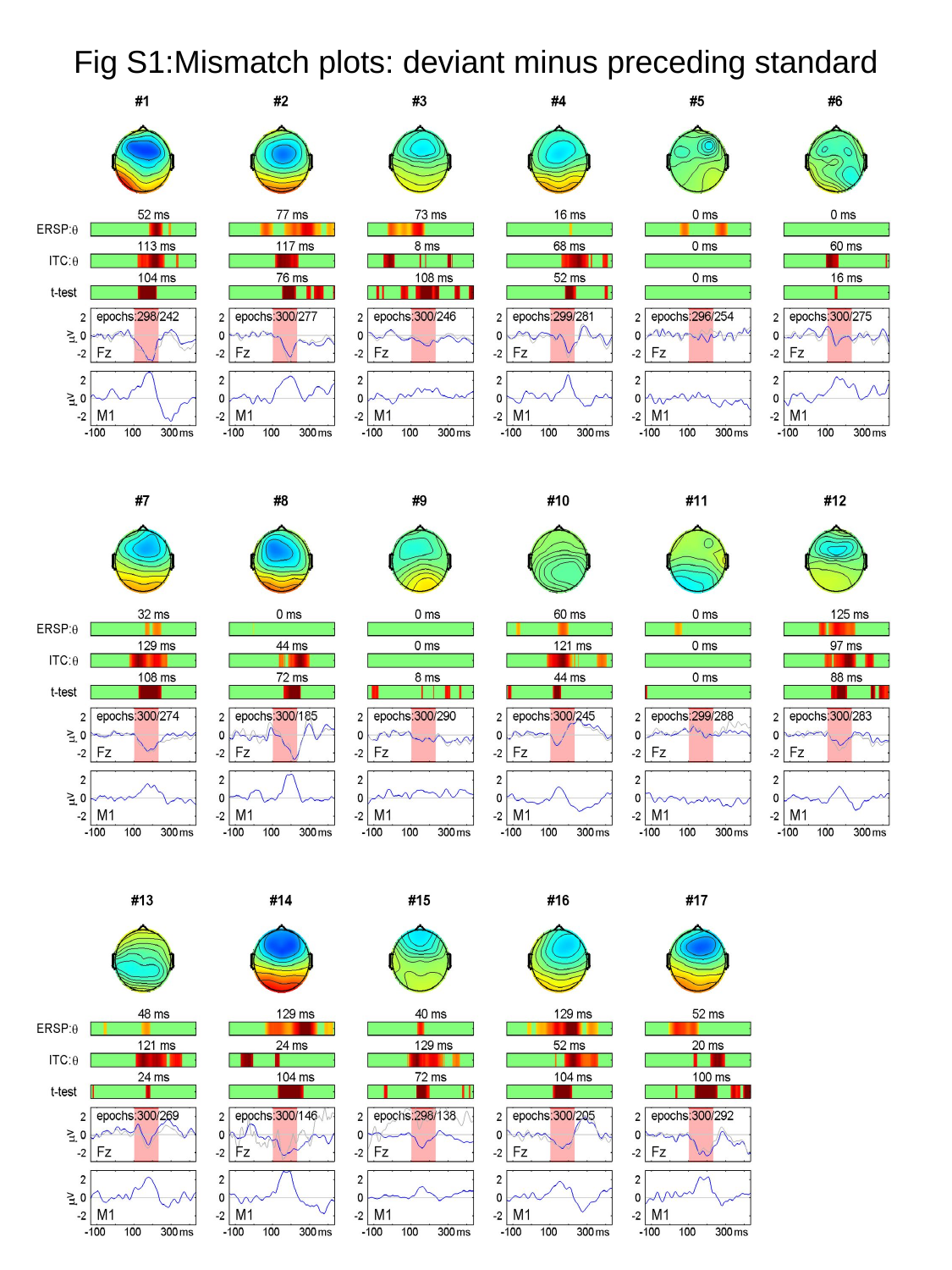

Fig S1:Mismatch plots: deviant minus preceding standard

## Slide 2
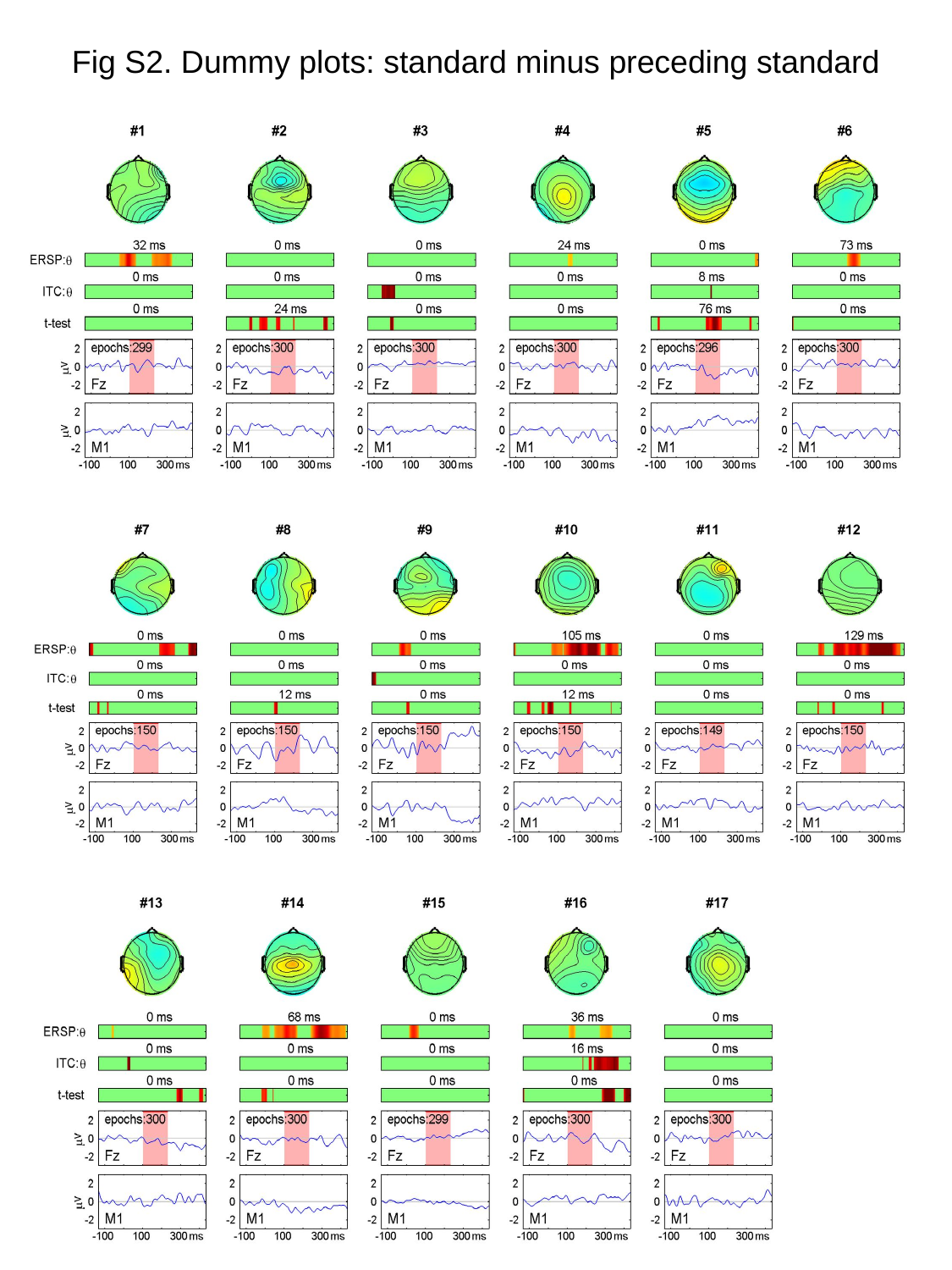

Fig S2. Dummy plots: standard minus preceding standard

## Slide 3
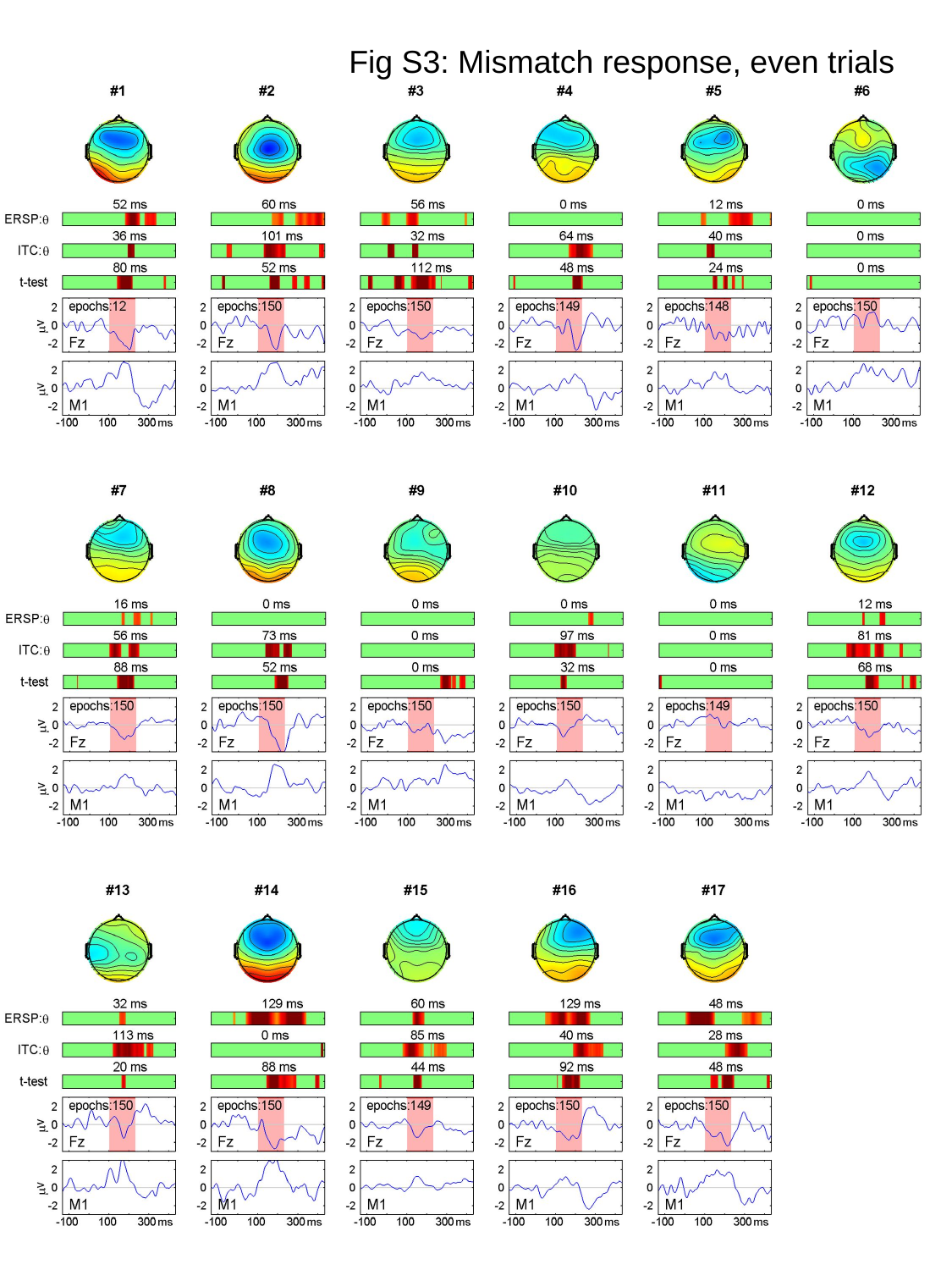

Fig S3: Mismatch response, even trials

## Slide 4
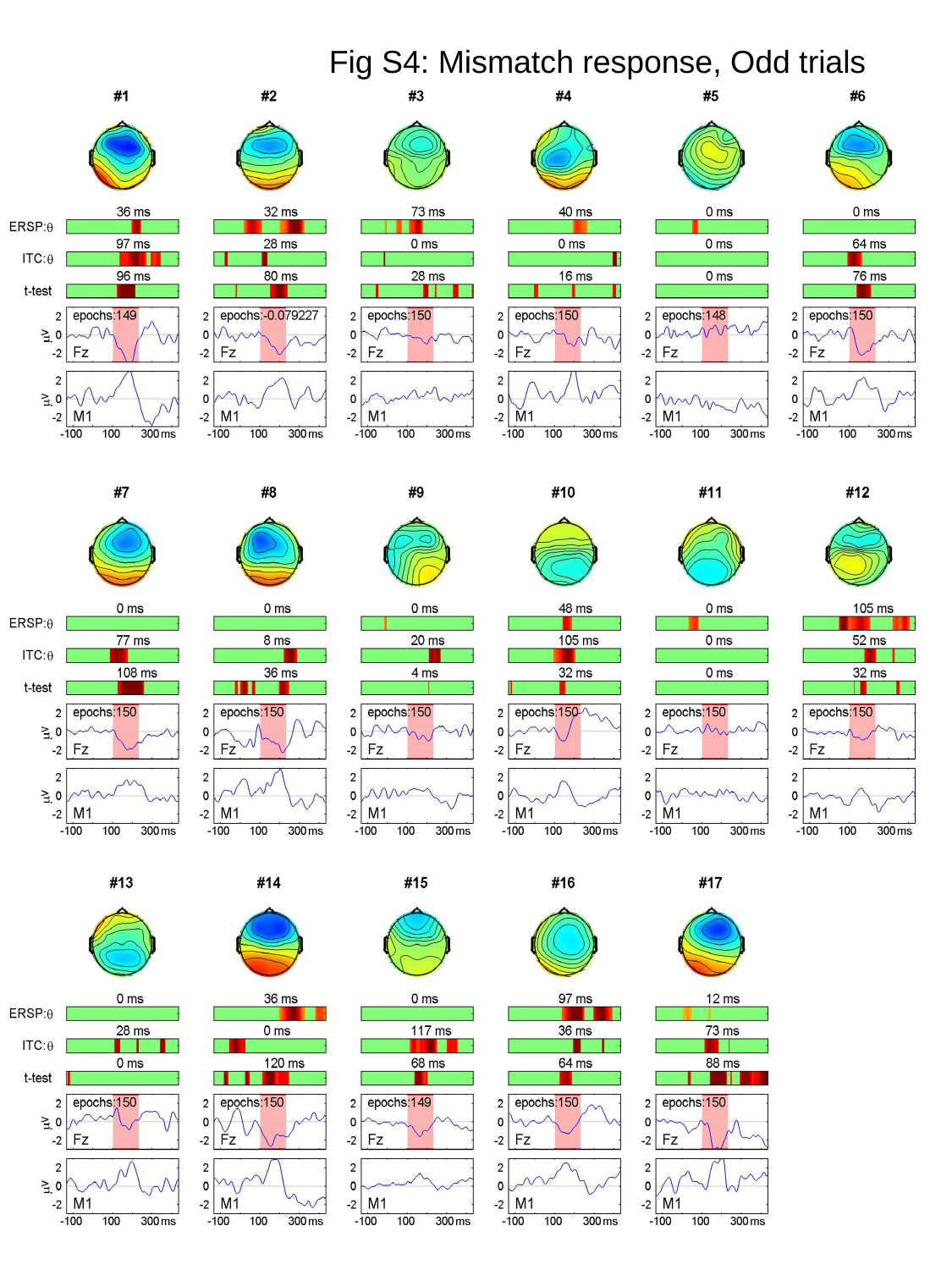

Fig S4: Mismatch response, Odd trials
